# The seroprevalence of cytomegalovirus infection in Belgium anno 2002 and 2006: a comparative analysis with hepatitis A virus seroprevalence

**DOI:** 10.1017/S0950268819000487

**Published:** 2019-03-18

**Authors:** G. S. A. Smit, S. Abrams, P. Dorny, N. Speybroeck, B. Devleesschauwer, V. Hutse, H. Jansens, H. Theeten, P. Beutels, N. Hens

**Affiliations:** 1 Department of Virology, Parasitology and Immunology, Faculty of Veterinary Medicine, Ghent University, Merelbeke, Belgium; 2 Department of Biomedical Sciences, Institute of Tropical Medicine (ITM), Antwerp, Belgium; 3 Institute of Health and Society (IRSS), Université catholique de Louvain, Brussels, Belgium; 4 Interuniversity Institute for Biostatistics and Statistical Bioinformatics, Hasselt University, Diepenbeek, Belgium; 5 Department of Epidemiology and Social Medicine, University of Antwerp, Antwerp, Belgium; 6 Department of Epidemiology and Public Health, sciensano, Brussels, Belgium; 7 Department of Veterinary Public Health and Food Safety, Faculty of Veterinary Medicine, Ghent University, Merelbeke, Belgium; 8 Scientific Directorate Infectious Diseases in Humans, Service of Viral Diseases, Sciensano, Brussels, Belgium; 9 Department of Laboratory Medicine, Antwerp University Hospital, Edegem, Belgium; 10 Centre for Health Economics Research and Modelling Infectious Diseases (CHERMID), Vaccine and Infectious Disease Institute, University of Antwerp, Antwerp, Belgium; 11 Center for the Evaluation of Vaccination, Vaccine and Infectious Disease Institute, University of Antwerp, Antwerp, Belgium; 12 School of Public Health and Community Medicine, The University of New South Wales, Sydney, Australia

**Keywords:** Cytomegalovirus, hepatitis A virus, estimating age and birth cohort-specific seroprevalence, seroincidence, mixture modelling

## Abstract

Cytomegalovirus (CMV) infection is endemic worldwide but its seroprevalence varies widely. The goal of this study was to estimate the age-specific seroprevalence of CMV infection in Belgium based on two cross-sectional serological datasets from 2002 and 2006. The seroprevalence was estimated relying on diagnostic test results based on cut-off values pre-specified by the manufacturers of the tests as well as relying on mixture models applied to continuous pathogen-specific immunoglobulin G antibody titre concentrations. The age-specific seroprevalence of hepatitis A virus (HAV), based on three Belgian cross-sectional serological datasets from 1993, 2002 and 2006, was used as a comparator since individuals acquire lifelong immunity upon recovery, implying an increasing seroprevalence with age. The age group weighted overall CMV seroprevalence derived from the mixture model was 32% (95% confidence interval (CI) 31–34%) in 2002 and 31% (95% CI 30–32%) in 2006. We demonstrated that CMV epidemiology differs from the immunizing infection HAV. This was the first large-scale study of CMV and HAV serial datasets in Belgium, estimating seroprevalence specified by age and birth cohort.

## Introduction

Cytomegalovirus (CMV) is a herpes virus that spreads mainly via close contacts through infected bodily fluids such as urine, blood, saliva or genital secretions [1]. The CMV seroprevalence varies globally and ranges approximately between 40% and 100% in adolescents and adults [2]. In immunocompetent children and adults, CMV infection remains usually asymptomatic, although it can cause mononucleosis-like symptoms and can occasionally lead to severe disease. By contrast, in immunocompromised patients, CMV infection is likely to cause significant morbidity and mortality and in pregnant women it may infect the unborn child through the placenta [3]. Such a congenital CMV (cCMV) infection is usually asymptomatic but may also lead to lifelong disabilities and even foetal or neonatal death [4]. Although several CMV vaccine candidates are under development, there is currently no licensed CMV vaccine available on the market [5].

Hepatitis A virus (HAV) is mainly transmitted through the faecal–oral route by ingestion of contaminated water or food, although close contact person-to-person transmission may also occur via this route, as well as via blood. Age-dependent HAV seroprevalence varies geographically and improvements in socio-economic, hygienic and food-safety conditions and to a lesser extent, active and passive immunisation activities, reduced the HAV force of infection, and thus lowered the overall seroprevalence over time [6, 7, 8]. HAV infection is usually symptomatic in adults and asymptomatic in young children (<5 years of age). Symptomatic hepatitis A infection may exceptionally lead to fulminant hepatitis and death. These severe manifestations are also more likely in adulthood than in childhood. The World Health Organization (WHO) emphasises that in countries with low and very low hepatitis A endemicity, vaccination should be targeted primarily to the groups at increased risk of hepatitis A [9]. Currently available inactivated HAV vaccines have been shown to provide long-term protection, with antibody persistence for 20 years after vaccination in more than 97% of vaccine recipients. Mathematical models predict 30 years of antibody persistence in over 95% of vaccine recipients [10, 11].

After primary infection with CMV or HAV, individuals acquire immunoglobulin G (IgG) antibodies against these pathogens. For HAV, IgG can also be vaccine-induced. Since the accumulated exposure time increases with age and the presence of IgG antibodies indicates past infection, the seroprevalence of IgG is expected to be monotonically increasing with age, provided that there is lifelong humoral immunity, time equilibrium at the disease (endemic equilibrium) and population level (demographic equilibrium), and that mortality attributable to infection can be ignored [12, 13].

Since HAV IgG antibodies are a marker of humoral immunity and assumed to be persisting through life [7, 14], HAV infections can be referred to as immunizing and conferring lifelong humoral immunity. In contrast, it was demonstrated that reactivation may contribute significantly to the transmission dynamics of CMV [15]. Primary infection is followed by latent infection that can reactivate, but also reinfection with a different CMV strain can occur, independent of anti-CMV IgG presence. These recurrent infections may also cause cCMV infection [16]. We will therefore refer to CMV as a non-immunizing infection throughout this manuscript.

Belgium has a total population of about 11 million [17] divided over the regions of Flanders, Wallonia and Brussels. An HAV seroprevalence of 55.1% (95% confidence interval (CI) 53.5–56.7%) was reported in 1993 in Flanders [18]. In 2013, a CMV seroprevalence in women of childbearing age of 41% (95% uncertainty interval (UI) 28–55%) and a cCMV birth prevalence of 52 per 10 000 live births (95% UI 41–64) was estimated in Belgium [19]. However, these authors also observed that no age-stratified seroprevalence reports were available at that time, that the coverage of the studies over Belgium was uneven, and that these studies showed differences in overall seroprevalence.

Information regarding the epidemiology and burden of infections is vital for evidence-based health policy. In this context, the goal of this study was to estimate the age-specific seroprevalence of CMV infection in Belgium based on two cross-sectional serological datasets from 2002 and 2006. In order to classify the individuals as seropositive or seronegative, we used both an approach based on cut-off values specified by the manufacturers of the diagnostic tests and a mixture model approach for the continuous antibody levels [20]. Due to its immunizing infection dynamics, the age-specific seroprevalence of HAV, based on three cross-sectional serological datasets from 1993, 2002 and 2006, was considered an excellent comparator for CMV seroprevalence. To better understand further differences between the seroprevalence profiles, we also estimated and compared the CMV and HAV seroincidence.

## Methods

### Study population and serological diagnostics

CMV cross-sectional samples were collected in 2002 and 2006, and examined for the presence of IgG antibodies using two different tests. Similarly, for HAV this was done in 1993, 2002 and 2006, and analysed for total (in 1993 and 2002) or IgG antibodies (in 2006) with three different diagnostic tests. The first set of samples was collected in Belgian hospitals in the context of baseline HAV, hepatitis B virus and hepatitis C virus prevalence assessment, cryopreserved and tested for HAV by Sciensano, the Belgian institute for health [18]. The second set was collected in 2002 in the context of the European Sero-Epidemiology Network (ESEN2) project in November 2001–February 2003, and samples were stored at −80 °C and analysed with the Enzygnost Anti CMV/IgG assay (Dade Behring, Germany) and the ETI-AB-HAVK PLUS (DiaSorin, Italy) test for HAV at Sciensano in 2009 [21]. The third set of samples was collected in January 2006–October 2007 to assess seroprevalence of vaccine-preventable diseases in Belgium, stored at −40 °C and analysed in 2013 with the CMV IgG assay for Elecsys and cobas e analysers (Cobas) and the anti-HAV IgG assay for Elecsys and cobas e analysers at the Laboratory for Medical Microbiology at the University Hospital of Antwerp [22]. Serum samples in 2002 and 2006 were collected from residual blood samples taken at a hospital visit or stay for individuals under 20 years of age or from donated blood for individuals of or over 20 years of age. Testing for CMV and HAV followed testing of among others measles, mumps, rubella, varicella-zoster virus (2002) and parvovirus B19 (2002). The majority of samples collected in 2002 and 2006 were tested for both CMV and HAV, but in some samples, there was not enough serum left to test both. Antibody levels were expressed in titres according to the manufacturers’ instructions. Data for HAV represent both natural and vaccine-induced antibodies because the serological tests used cannot distinguish between both. No information was available regarding vaccine uptake. The protocols for the 1993, 2002 and 2006 sero-surveys were approved by the ethical review board of the University of Antwerp and the University Hospital of Antwerp.

### Statistical analysis

We used the cross-sectional serological data to estimate the age-specific seroprevalence, denoted by *π* (*a*). Since maternal antibodies can persist for a few months after birth, we excluded serological data obtained for children aged <1 year. The fixed diagnostic cut-off value provided by the producer of the assay was used to divide a single population in seronegative (*δ*_*i*_ = 0; susceptible) and seropositive (*δ*_*i*_ = 1; infected/recovered) individuals (with humoral immunity exceeding the threshold, implying past infection or vaccination). Hence, the immunological status of the individual, say Δ_*i*_, conditional on the individual's age *a*_*i*_ follows a Bernoulli distribution, i.e. 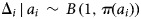. Seroprevalence by age and birth cohort was estimated for the different study years using flexible spline models; more specifically thin plate regression splines [23, 24]. A weighted mean over age was used to compare the mean overall seroprevalence and subsequent 95% CI over the different methods and years. A few (0.65%) equivocal results in the CMV 2006 data were excluded from the analysis.

Alternatively, it is possible to model the observed continuous antibody levels directly without the specification of (subjective) cut-off values, thereby avoiding misclassification of individuals as seropositive or seronegative. In order to do so, one can use mixture modelling [20, 25]. We used a mixture model with two mixture components where the log-transformed antibody concentration *Y*_*i*_, *i* = 1, …, *n*, for the *i*th subject in the sample conditional on his/her age *a*_*i*_ has distribution given by:
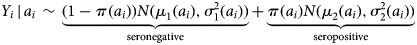


The probabilities (1 − *π* (*a*_*i*_)) and *π* (*a*_*i*_) are the age-dependent mixture probabilities, for which *π* (*a*_*i*_) can be interpreted as the seroprevalence at age *a*_*i*_ (4-year age groups). Furthermore, *μ*_*j*_(*a_i_*) and 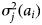, *j* = 1, 2, are the mean and the variance of log-transformed antibody concentrations in the susceptible (*j* = 1) and infected (*j* = 2) components at age (*a_i_*) (4-year age groups) [20]. Label switching was prevented by using a shift parameter for the means. We used the maximum likelihood estimation approach and hence calculated the relevant log-likelihood and then minimised the negative log-likelihood with general-purpose optimisation in R version 3.4.2 [26] to estimate the model parameters. The likelihood was adjusted to account for left- and right-censored data. Latin hypercube sampling was used to find a suitable set of starting parameters. The goodness-of-fit was evaluated by visual inspection, including of the density functions. The univariate delta method was used to calculate the variance of the seroprevalence and subsequent 95% CI [27]. A weighted mean over the age groups was used to compare the overall seroprevalence and subsequent 95% CI over the different methods and years.

The effect of time was assessed by calculating the estimated seroincidence by subtracting the estimated seroprevalence in 2002 (1993) from the seroprevalence in 2006 (2002) in all birth cohorts. The multivariate delta method was used to calculate the variance of the seroincidence and subsequent 95% CI. The overall and age group-specific effect of gender on seroprevalence was assessed by comparing males and females while using the multivariate delta method to calculate the variance and subsequent 95% CI [27].

Since diagnostic assays differed between 2002 and 2006, a random subset of the 2002 samples was re-tested with the diagnostic assay used for the 2006 samples. This allowed us to investigate the influence of using these different assays. We assessed the possibility of transforming the CMV 2002 quantitative data to match the 2006 data using the censored regression model censReg in R with exclusion of the censored susceptible component. The seroprevalence and seroincidence using the transformed 2002 data were calculated using the mixture model in the same manner as described above. Furthermore, we assessed if an association could be found between HAV and CMV antibody titres in both 2002 and 2006 using scatterplots.

Statistical uncertainty about the seroprevalence and seroincidence was quantified as mentioned above and summarised by the mean and 95% CI and all calculations were performed using R version 3.4.2 [26].

## Results

### Cytomegalovirus

In total, 2915 and 1683 serological samples were analysed for CMV-specific IgG, in 2002 and 2006, respectively, which included 1492 and 837 samples from females and 1423 and 846 from males, respectively. In 2002, 1943 samples were left-censored with a titre of 50 as lower limit on the original scale. In 2006, 1088 samples were left-censored at 0.15 U/ml and 142 samples right-censored at 500 U/ml on the original scale. The mean age of the population sample was 26 years (95% CI 2.0–64; range 1–72) in 2002 and 29 years (95% CI 2.0–62; range 1–65) in 2006. We did not observe large differences when we transformed the 2002 data to match the 2006 data, indicating negligible influence of the diagnostic test differences.

**Fig. 1.**
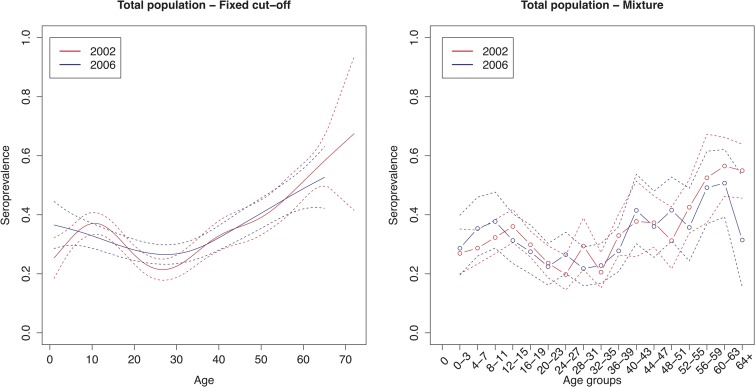
Cytomegalovirus seroprevalence in function of age in 2002 and 2006 estimated by using splines and fixed cut-offs and by the mixture model. *Note*: Age groups consist of 1 year in the spline fit and 4 years in the mixture model.

**Fig. 2.**
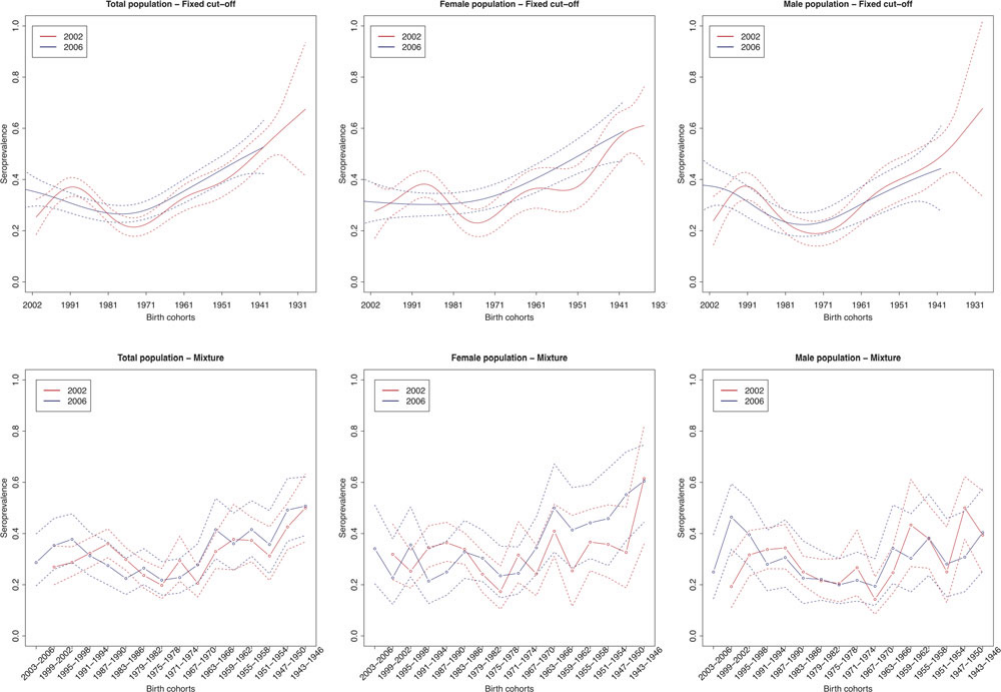
Cytomegalovirus seroprevalence by birth cohort in 2002 and 2006 estimated by splines and fixed cut-offs and by the mixture models (rows) for the total population, females and males (columns). *Note*: Birth cohorts consist of 1 year in the spline fit and 4 years with in the mixture model.

The age group weighted overall CMV seroprevalence estimated by the mixture model was 32% (95% CI 31–34%) in 2002 and 31% (95% CI 30–32%) in 2006. A similar age-weighted overall seroprevalence was estimated using the spline fit (33%; 95% CI 32–34% in 2002 and 2006). Figure 1 shows the spline fits of the CMV seroprevalence in function of age in 2002 and 2006 using the fixed cut-offs (left panel) and graphically depicts the seroprevalence per age group estimated by the mixture model (right panel). At one year of age the spline fit showed a seroprevalence of 24% (95% CI 16–32%) in 2002 and 37% (95% CI 28–46%) in 2006. A decrease in seroprevalence was observed around the age range 12–23 years in 2002 followed by a slow increase reaching 50% around the age of 56 years. In 2006, a similar but less pronounced decrease followed by a similar increase reaching 50% around the age of 60 years was seen. Figure 2 shows the seroprevalence of CMV per birth cohort in the total population (left column), women (middle column) and men (right column), separately. The figure depicts the spline fits of the CMV seroprevalence in function of birth cohorts by using the fixed cut-offs (upper row) and by the mixture model (lower row).

**Fig. 3.**
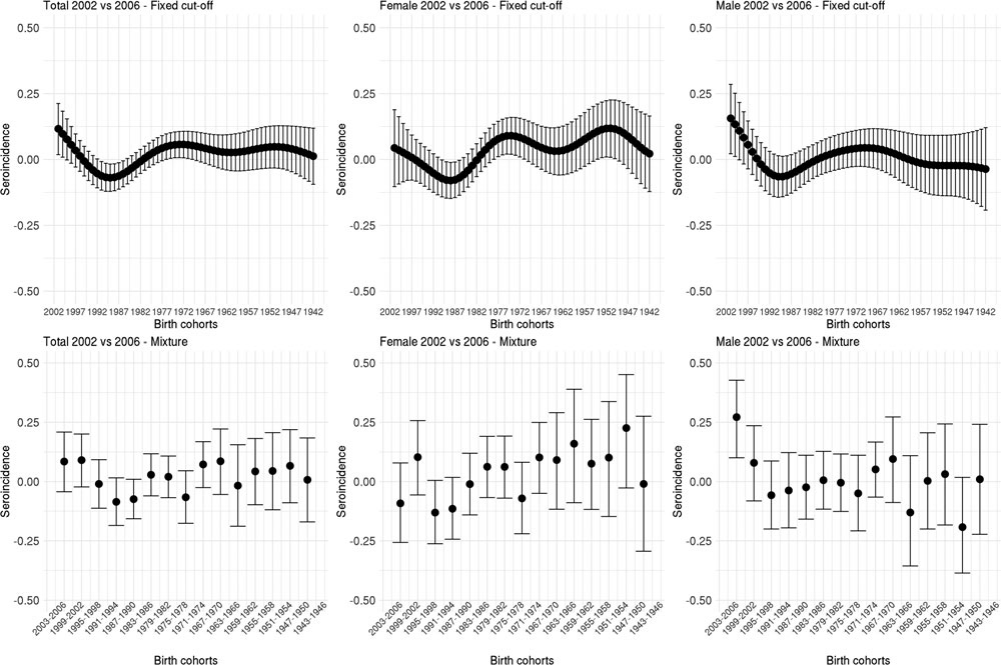
Cytomegalovirus seroincidence by birth cohort in 2006 compared with 2002 estimated using splines and fixed cut-offs and by the mixture models (rows) for the total population, males and females (columns). *Note*: Birth cohorts consist of 1 year in the spline fit and 4 years in the mixture model.

Figure 3 shows the estimated seroincidence per birth cohort for the total population (left panels), females (middle panels) and males (right panels). We compared the 2002 seroprevalence with that of 2006 per birth cohort, first using the fixed cut-off approach (upper row). The seroincidence increased in people born after 1999, decreased in people born in the period 1985–1992 and increased in people born in 1969–1977. There were gender-related differences. Females born after 1999 showed no increase in seroincidence (whereas males did). Seroincidence decreased in females born 1985–1991, and increased in females born 1970–1979 and 1949–1955. The mixture model approach (lower row) showed only a significantly increased seroincidence in males born in 1999–2002, despite it displaying overall similar patterns (including decreased seroincidence in females born 1987–1994).

For women of childbearing age (16–47 years of age) we estimated an age group weighted seroprevalence of 30% (95% CI 29–30%) in 2002 and 31% (95% CI 30–31%) in 2006. A significantly higher seroprevalence was found in women compared with men in the age groups 16–19 and 36–39 years of age in 2002. In 2006, a significantly higher seroprevalence was found in women compared with men in general. A significantly lower seroprevalence was found in women compared with men in the age group 4–7 years of age and a significantly higher seroprevalence was found in the age groups 36–39 and 64+ years in 2006. The age-specific CMV seroprevalence per region at the different time points can be found in Supplementary Figure S2.

### Hepatitis A virus

In 1993, 2002 and 2006, respectively, 3792, 3350 and 1620 samples were tested for HAV antibodies. These samples consisted of 1780, 1689 and 811 samples from females and 2012, 1661 and 809 samples from males in 1993, 2002 and 2006, respectively. The mean age in the sampling populations was 40 years (95% CI 5.0–81; range 1–100) in 1993, 23 years (95% CI 2.0–64; range 1–72) in 2002 and 30 years (95% CI 2.0–63; range 1–65) in 2006. In 2002, 2906 samples (95.6% of CMV samples and 86.7% of HAV samples), and in 2006, 1609 samples (99.7% of CMV samples and 99.3% of HAV samples) were tested for both CMV and HAV. We did not observe an association between HAV and CMV antibody titres in both 2002 and 2006 (Supplementary Figure S1). Unfortunately, we were not able to use a mixture model to estimate the seroprevalence of HAV due to excessive censoring of the continuous antibody titre levels.

**Fig. 4.**
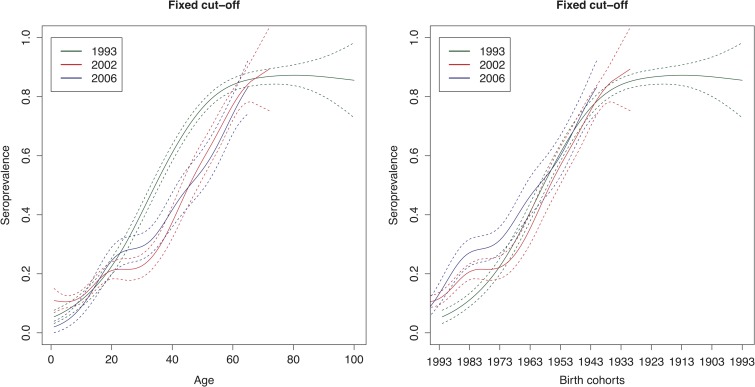
Seroprevalence of hepatitis A virus antibodies as a function of age and birth cohorts in 1993, 2002 and 2006 resulting from primary infection or vaccination using splines and the fixed cut-offs.

The age-weighted overall HAV seroprevalence using the spline fit was 54% (95% CI 38–70%) in 1993, 26% (95% CI 18–33%) in 2002 and 31% (95% CI 23–39%) in 2006 in Belgium. Figure 4 shows that the estimated HAV seroprevalence increases with age, with low seroprevalence in young and middle-aged adults and high seroprevalence in the elderly. In 1993, the seroprevalence reached 50% in people around 35 years of age, whereas in 2002 and 2006, 50% seroprevalence was only reached around the age of 45 years.

**Fig. 5.**
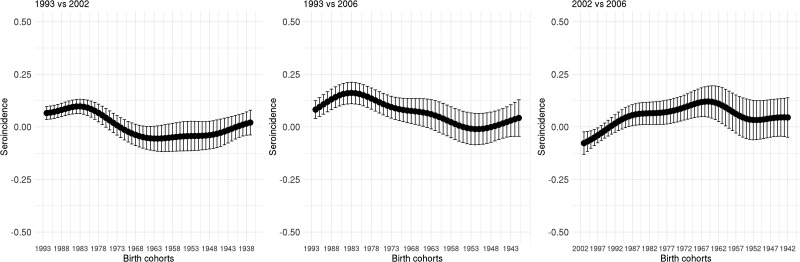
Seroincidence of hepatitis A virus antibodies per birth cohort comparing 2002–1993, 2006–1993 and 2006–2002 estimated using splines and the fixed cut-offs.

Figure 5 shows the seroincidence per birth cohort for 2002 compared with 1993 (left panel), 2006 compared with 1993 (middle panel) and 2006 compared with 2002 (right panel). It shows that persons born before 1976 did not gain any noticeable immunity against HAV between 1993 and 2002 and persons born before 1964 between 1993 and 2006. In contrast, younger persons have, on average, gained non-negligible immunity, either through natural infection or through vaccination. A negative seroincidence could be observed when we compared the 2002 and 2006 data from people born after 1996, whereas people born 1959–1989 gained non-negligible immunity. The age-specific HAV seroprevalence per region at the different time points can be found in Supplementary Figure S3.

## Discussion

The seroprevalence of CMV infection was estimated either by using a fixed cut-off approach or by considering a mixture model for continuous antibody titre concentrations. In addition, CMV infection dynamics were assessed by studying serial serology of CMV and HAV. Unfortunately, we were unable to estimate the seroprevalence and seroincidence of HAV with a mixture model due to excessive censoring. This is unfortunate especially since it has been shown that the antibody response to natural infection is stronger than to vaccination. Vaccine-induced antibody levels might wane over time, which makes setting appropriate cut-off values for diagnostic tests difficult [28]. The main advantages of using a mixture model are that all information is used on the original (or transformed) continuous scale of antibody activity levels and that observations are assumed to originate from two (or more) distributions, referring to a susceptible and an infected subpopulation (among other possible subpopulations), with different distributional parameters. This implies that the somewhat subjective cut-offs provided by the manufacturer, usually chosen on clinical diagnostic or screening grounds, do not have to be used. Next to the difficulties encountered with setting meaningful cut-off values to continuous distributions, the choice of cut-offs also depends on the aim of the test. From an epidemiological perspective, the seroprevalence in the population at each age, not the detection of clinical significant antibody levels in an individual, is the primary consideration, which makes the mixture model a more appropriate method [20, 29, 30].

The age group-weighted overall CMV seroprevalence estimated by the mixture model was found to be 32% (95% CI 31–34%) in 2002 and 31% (95% CI 30–32%) in 2006. This is low compared with seroprevalances found in other countries and in Belgium. The systematic review and random-effects meta-regression by Zuhair *et al*. [2] estimated a mean seroprevalence in the general population of 83% (95% UI 78–88%) globally, with the lowest in the European region (66% (95% UI 56–74%)) and Ireland (39% (95% UI 18–62%)) in contrast to the highest in the Eastern Mediterranean region (90% (95% UI 85–94%)) and Turkey (96% (95% UI 93–98%)). For women of childbearing age (16–47 years of age), we estimated an age group-weighted seroprevalence of 30% (95% CI 29–30%) in 2002 and 31% (95% CI 30–31%) in 2006, which is lower compared with the meta-analysis-based estimate for Belgium of 41% (95% UI 28–55%) by Smit *et al*. [19].

Whilst seroprevalence estimates in the mixture model closely reflected those using the fixed cut-off approach, the main difference between the two methods was found in the peaks observed in the age group 40–43 years for both 2002 and 2006 in the mixture model. Both methods showed that a sizable fraction was infected before one year of age and that the CMV seroprevalence in 2002 and 2006 showed a ‘dip’ in the age range 12–39 years followed by a slow increase reaching 50% around the age of 56 years in 2002 and 60 years in 2006. The decrease in seroprevalence might be explained by the sampling method, which included two groups: individuals under 20 years who visited the hospital for diagnosis or screening and blood donors of and over 20 years of age. This seems plausible since the HAV data in 2002 and 2006 had a similar sampling scheme and showed a similar ‘dip’ between approximately 20 and 35 years of age, in contrast to the HAV data from 1993, which were based on hospital samples. These two groups, hospital patients and blood donors, might differ in health status, since blood donors are screened for some infectious diseases and excluded when found at risk or positive. Other factors might be of influence as well, such as socio-economic status, with lower levels being associated with higher seroprevalence [31].

The spline fit and the mixture model showed an increased seroincidence in males born before 1999 (2 years of age in 2002 and 6 in 2006) and additionally in the total population with the fixed cut-off approach. Interestingly, both methods (although not significant in the mixture model) showed a decreased seroincidence in females and the total population born approximately between 1985 and 1991 (11–17 years of age in 2002 and 15–21 in 2006). We cannot fully link this phenomenon to the sampling method (samples from individuals visiting the hospital under 20 years and from blood donors of and over 20 years of age), since if this were the case we would suspect the negative seroincidence to be present in the birth years 1983–1986 (16–19 years of age in 2002 and 20–23 in 2006). Next to the possibility of an artefact or confounding factors, this might also indicate a violation of the assumptions of lifelong immunity; time equilibrium at the disease and/or population level; or that mortality attributable to CMV infection can be ignored. A similar dip in the seroprevalence fit at a similar age was shown by Goeyvaerts *et al*. [12] for human parvovirus B19, which made them question and explore the assumption of lifelong immunity. We cannot rule out a cohort effect due to a demographical shift or CMV related mortality. However, it has been shown that the virus can reactivate and reinfection with a different CMV strain can occur [16]. This may imply that the assumption of primary infection followed by lifelong immunity, including IgG-positive antibody levels, might not hold and that boosting through reactivation and reinfection is likely.

Gaining insight in the processes underlying CMV infection dynamics is of major public health interest especially since an increase in IgG seroprevalence between 2002 and 2006 was observed in the child-bearing age category of the total population and females specifically. The risk in pregnant women might then be underestimated. This increased seroincidence was seen in the spline fit in females and the total population born between approximately 1970 and 1977 (25–32 years of age in 2002 and 29–36 in 2006) and in females born between 1949 and 1955 (48–52 years of age in 2002 and 52–56 in 2006). Furthermore, we noticed a higher seroprevalence in women compared with men between 36 and 39 years of age, which might indicate a faster increase in seroprevalence in recent mothers (having young children in the household).

Although not directly comparable, a similar difference between men and women was seen in the NHANES study in the USA [32]. In the Netherlands, van Boven *et al*. [15] merged a statistical analysis with transmission models and described three distributions of antibody measurements: low, intermediate and high antibody concentrations, which were related to the uninfected, latently infected and latently infected after reactivation or reinfection groups, respectively. In contrast to other infectious diseases, e.g. as seen for HAV [33], the Dutch CMV seroprevalence increased gradually with age. Their findings indicated that the transmissibility of primary infection is much lower than the transmissibility after reactivation and add to the belief that transmission from adults after infectious reactivation is an important driver of CMV transmission in the population. Noteworthy is that they found a higher incidence of infection in adult women than in adult men of similar age [15]. However, the possibility of antibody waning has not been observed nor tested yet. The majority of studies show a gradual increase in seroprevalence with age [31] although there seem to be studies that might have similar seroprevalence patterns compared with our data (see, e.g. Barbi *et al*. [34]; de Ory *et al*. [35]; Wentworth *et al*. [36]). We cannot directly compare the methodology and results of the studies described above with our study, yet our data at least show that CMV infection dynamics do not follow the same pattern as we observe for HAV.

Similarly to what has been reported by Beutels *et al*. [18] and Kurkela *et al*. [7], HAV antibody presence increased with age, with low seroprevalence in young and middle-aged adults and high seroprevalence in the elderly, indicative of historic transmission. The HAV seroprofile in birth cohorts to some extent appears to reflect that endemic circulation seemed to be discontinued around the 1970s, potentially due to changes in hygienic and socio-economic conditions over time [7, 33].

An increased seroincidence was observed in the birth cohorts born after 1976 in 2002 (26 years of age in 2002), as already noted by Beutels *et al*. [33], and 1964 in 2006 (42 years of age in 2006) compared with the set of samples in 1993. People born between 1959 and 1989 (17–47 years of age in 2006) gained non-negligible immunity when we compared the 2002 and 2006 datasets. This might be explained, at least partly, by outbreaks [37] and by vaccination of high-risk groups (including, e.g. health care workers, travellers to endemic regions and people with haemophilia) and by the fact that vaccination was used as outbreak control measure [7] from 1992 onwards in Belgium. It was estimated that 708 095 people between 1995 and 2006 (or 6.7% of all Belgian residents) received a full HAV vaccine schedule in Belgium [33]. In contrast, a negative seroincidence could be observed when we compared the 2002 and 2006 data from people born after 1996 (10 years of age in 2006). This might be explained by the detection of total antibodies in 2002 in contrast to IgG antibodies in 2006. The high susceptibility to HAV in young and middle-aged adults demonstrates that it might be beneficial to continue vaccination of risk groups in Belgium. Children between 1 and 12 years of age travelling to high endemic regions were recommended for targeted vaccination by Beutels *et al*. [33], since these children can be an important source for outbreaks when they return with asymptomatic HAV infection in a society with increasing proportions of susceptible adults.

The strengths of this study were the availability of serial serological data with age specification; the possibility of comparison between the CMV infection dynamics and the immunizing HAV infection dynamics; and the use of a mixture model. The limitations, however, were the censoring in the serological data; and the comparability between the different datasets and thus populations, based on the sampling scheme and the diagnostic methods used (e.g. the majority of samples from individuals under 20 years of age especially in 2002; datasets containing hospital samples of individuals under 20 years and samples of blood donors of and over 20 years of age in 2002 and 2006 and only hospital samples (mainly from Flanders) in 1993; different diagnostic assays used; and detection of total HAV antibodies in 1993 and 2002 in contrast to only HAV IgG in 2006). This study reinforces the importance of distinguishing between sampling sources.

We acknowledge that CMV and HAV are different viruses, yet our aim was to compare the seroprevalence profile over age of CMV with an infection known to confer lifelong immunity, implying in theory a monotonically increasing seroprevalence pattern over lifetime, which may not necessarily be observed in a cross-sectional population sample by age. In a potential comparison of CMV with measles, mumps or rubella, vaccination for the latter pathogens would play too much of a role. HAV vaccination was limited to approximately 7% of all Belgian residents [33], which is likely to be similar to the percentage for this dataset. Moreover, seroprevalence of both HAV and CMV is known to be related to socio-economic status [6, 7, 31, 33] indicating similarities in transmission.

We showed that the CMV infection dynamics do not follow the same patterns as those for HAV infections. To be able to explain the observed seroprevalence profile, a prospective cohort study and/or several serial studies are needed. The latter option should be population-based or at least include a head-to-head comparison to reveal the bias (e.g. hospital patient–age–year *vs.* blood donor–age–year). In the absence of such data, it might be beneficial to explore several immunological scenarios in mathematical models and infer waning and boosting rates using serological and social contact data. Similar studies were performed for human parvovirus B19 [12], measles [38], pertussis [39, 40] and CMV as described previously [15]. It would be of interest to study the possibility of antibody waning and multiple (re)infection and reactivation events in the case of CMV infections.

This was the first large-scale study of CMV and HAV serial serological survey datasets in Belgium, estimating age and birth cohort-specific seroprevalence and seroincidence. In addition, it highlights the benefits of using a mixture model for epidemiological purposes and that it is important to distinguish between sampling sources. We further demonstrated that the CMV epidemiology differs from that of an immunizing infection such as HAV. Good surveillance systems need to be maintained to monitor continuously changing trends, and to support the implementation of appropriate intervention and prevention policies.

## Supplementary material

For supplementary material accompanying this paper visit https://doi.org/10.1017/S0950268819000487. click here to view supplementary material
