# Supplementary material for: The seroprevalence of cytomegalovirus infection in Belgium anno 2002 and 2006: a comparative analysis with hepatitis A virus seroprevalence
Source: Epidemiol Infect. 2019 Mar 18;147:e154. doi: 10.1017/S0950268819000487 (PMC6518518; doi:10.1017/S0950268819000487)
Supplement: Supplementary file 1 [file S0950268819000487sup001.docx]

Epidemiology and Infection

Supplementary Material

# The seroprevalence of cytomegalovirus infection in Belgium anno 2002 and 2006: a comparative analysis with hepatitis A virus seroprevalence

G.S.A. Smit, S. Abrams, P. Dorny, N. Speybroeck, B. Devleesschauwer, V. Hutse, H. Jansens, H. Theeten, P. Beutels, N. Hens.

# Supplementary Material

In 2002, 2906 samples (95.6% of samples tested for cytomegalovirus (CMV) and 86.7% of samples tested for hepatitis A virus (HAV)) and in 2006 1609 samples (99.7% of CMV samples and 99.3% of HAV samples) were tested for both CMV and HAV. We assessed if a correlation could be found between HAV and CMV antibody titres within these samples that were tested for both HAV and CMV in both 2002 and 2006 using scatterplots. No correlation was found as shown in Supplementary Figure S1.

**
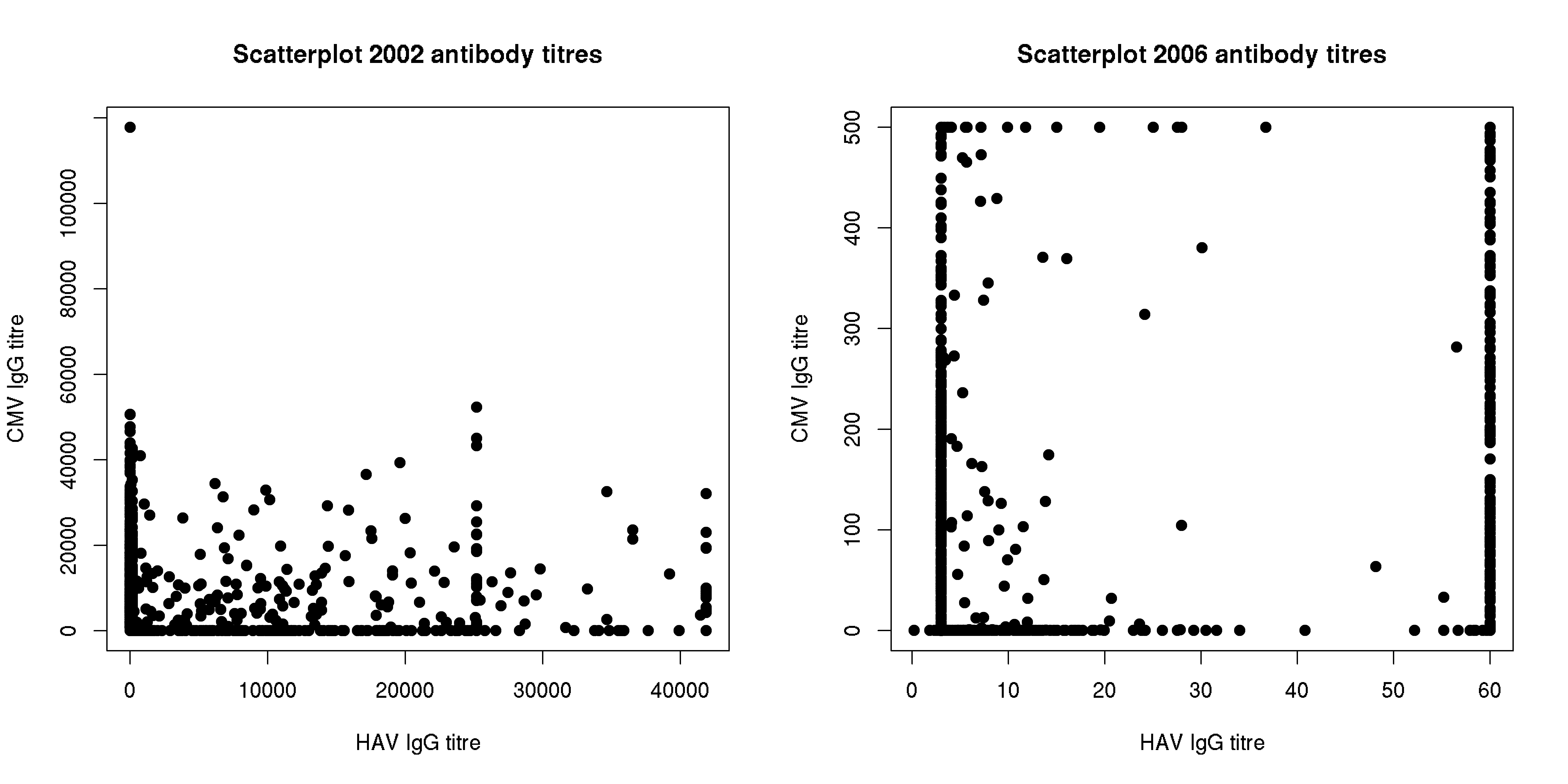
**

**Supplementary Fig S1. Scatterplot of hepatitis A virus and cytomegalovirus antibody titres in 2002 and 2006.**

In addition, we estimated the age-specific CMV and HAV seroprevalence per region at the different time points (Supplementary Figure S2 and S3).


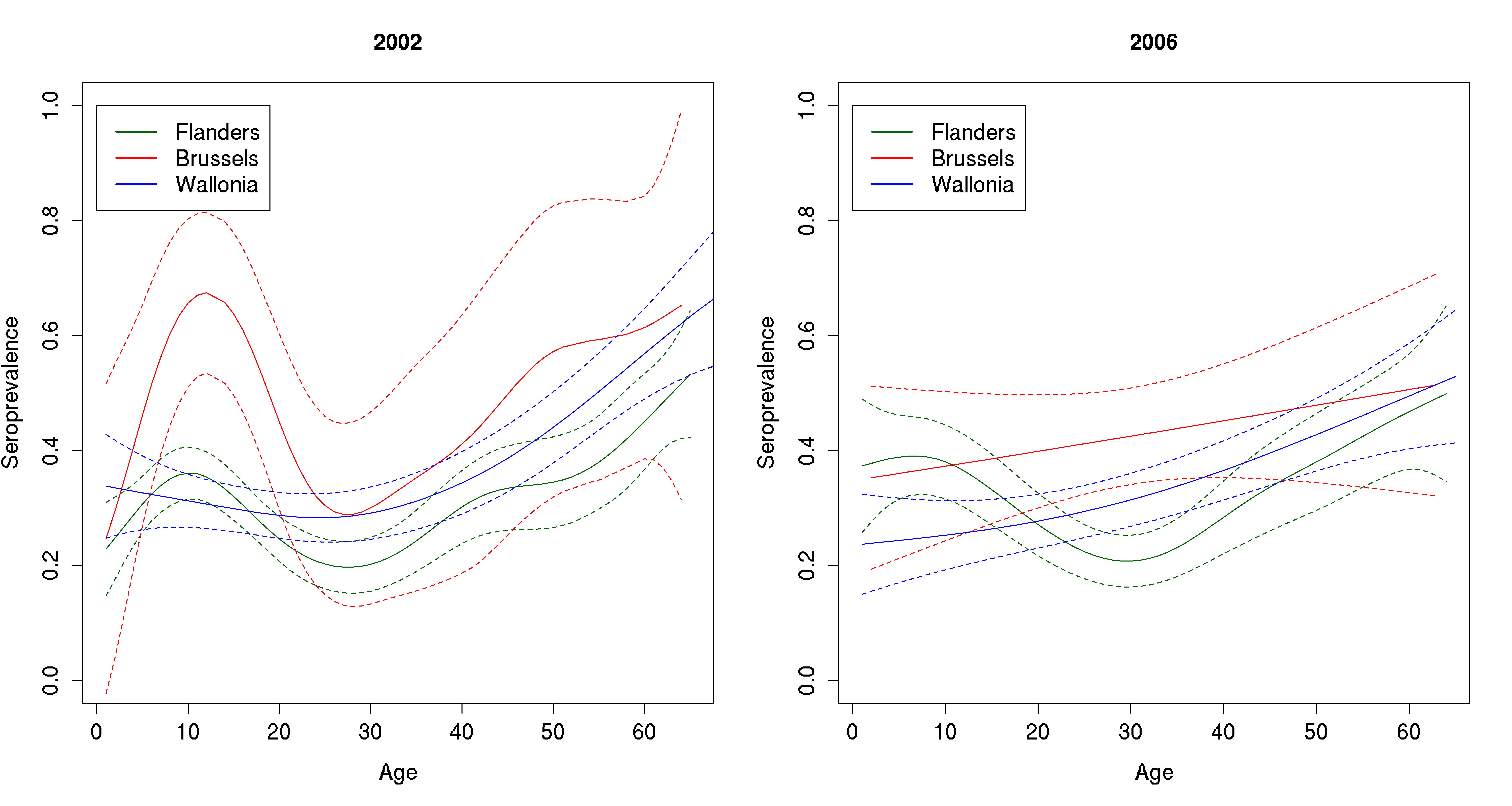


**Supplementary Fig S2. Age-specific cytomegalovirus seroprevalence by Belgian region in 2002 and 2006 estimated using splines and the fixed cut-offs.**

Note: There were relatively few samples for Brussels.





**Supplementary Fig S3. Age-specific hepatitis A virus antibodies seroprevalence per Belgian region in 1993, 2002 and 2006 resulting from primary infection or vaccination (in 2002 and 2006) estimated using splines and the fixed cut-offs.**

Note: There were relatively few samples for Brussels in all years and for Wallonia in 1993.
